# The role of nuclear technologies in the diagnosis and control of livestock diseases—a review

**DOI:** 10.1007/s11250-012-0077-5

**Published:** 2012-01-28

**Authors:** Gerrit J. Viljoen, Antony G. Luckins

**Affiliations:** Animal Production and Health Section, Joint FAO/IAEA Division, International Atomic Energy Agency, Vienna International Centre, P.O. Box 100, 1400 Vienna, Austria

**Keywords:** Nuclear techniques, Radiation attenuation, Radiolabeled probes, Radioimmunoassay, Disease diagnosis, Stable isotopes

## Abstract

Nuclear and nuclear-related technologies have played an important role in animal health, particularly in relation to disease diagnosis and characterization of pathogenic organisms. This review focuses primarily on how and where nuclear technologies, both non-isotopic and isotopic methods, have made their impact in the past and where it might be expected they could have an impact in the future. The review outlines the extensive use of radiation attenuation in attempts to create vaccines for a multiplicity of pathogenic organisms and how the technology is being re-examined in the light of recent advances in irradiation techniques and cryopreservation/lyophilization that might obviate some of the problems of maintenance of viable, attenuate vaccines and their transport and use in the field. This approach could be used for a number of parasitic diseases where vaccination has been problematic and where investigations into the development of molecular vaccines have still failed to deliver satisfactory candidates for generating protective immune responses. Irradiation of antigens or serum samples also has its uses in diagnosis, especially when the samples need to be transported across international boundaries, or when handling the pathogens in question when carrying out a test presents serious health hazards to laboratory personnel. The present-day extensive use of enzyme immunoassays and molecular methods (e.g., polymerase chain reaction) for diagnosis and characterization of animal pathogens has its origins in the use of isotope-labeled antigens and antibodies. These isotopic techniques that included the use of ^75^Se, ^32^P, ^125^I, and ^35^S isotopes enabled a level of sensitivity and specificity that was hitherto unrealized, and it is prescient to remind ourselves of just how successful these technologies were, in spite of their infrequent use nowadays. Finally, the review looks at the potential for stable isotope analysis for a variety of applications—in the tracking of animal migrations, where the migrant are potential carriers of transboundary animal diseases, and where it would be useful to determine the origins of the carrier, e.g., Highly Pathogenic Avian Influenza and its dissemination by wild water fowl. Other applications could be in monitoring sequestered microbial culture (e.g., rinderpest virus) where in the case of accidental or deliberate release of infective culture it would be possible to identify the laboratory from which the isolate originated.

## Introduction

The creation of the Joint FAO/IAEA Division in 1964 was a significant event in assisting developing countries to improve their livestock and agriculture to enable greater productivity and improved food security. Since that time, the Joint Division has sought to introduce innovative technologies, based on nuclear techniques that will allow farmers to provide safe, healthy food in often harsh environment and in the face of serious infectious diseases that compromise productivity. Among the earliest efforts in animal health specifically to seek answers to the problems caused by infectious diseases were the efforts to develop irradiated vaccines. Interest in this technology was eventually superseded by alternative technologies that were deemed likely to provide solutions more rapidly. These techniques, employing various molecular methods involving recombinant antigens, have not delivered as expected and attention is once again focusing on the potential of irradiated vaccines. Complementary to control of diseases is the need to be able to accurately identify infected animals quickly and simply. Nuclear techniques were in the forefront of a number of highly sensitive and specific tests for diagnosing infections. Although for the most part such tests using radioisotope labels have been replaced, the tests that have been developed rely in their inception on the radiolabeling methods that preceded them, so they are still very much nuclear-related. Opportunities have arisen recently to consider the use of stable isotope analysis in understanding the migration of wild birds and their involvement in the spread of Highly Pathogenic Avian Influenza, and these safe, non-radioactive isotopes may even be of use in diagnosis of disease or in typing pathogenic organisms. The latter could be of use when considering the storage and sequestration of pathogens, for use as a method of identifying the source of an accidental release, or a deliberate act of bioterrorism. This review highlights the various nuclear techniques that have been employed in the field of animal health and highlights the potential for further research and development.

### Radiation-attenuated vaccines and diseases of domesticated livestock

Vaccination is a cost-effective way of controlling animal disease. In the case of viral diseases, it might be the only way to control them successfully in the absence of alternative therapies. Bacterial and parasitic diseases can sometimes be controlled by antibiotics and chemotherapeutics, but these have their limitations, as reinfection can still occur, and it may be necessary to continually treat animals. Also, the increased incidence of antibiotic and drug resistance as well as the presence of residues in food for human consumption emphasizes the importance of seeking alternative methods of control. Hence, there is a strong argument to develop vaccines, especially for parasitic diseases, where presently long-term drug treatment, with all its potential problems, might be the only way to prevent disease. Although anti-viral and anti-bacterial vaccine development has been quite successful, this is not the case for vaccines against parasitic diseases and there are only a few commercially available products.

Gamma irradiation is one of the most effective means of sterilization, having been applied to ensure biological safety of food products, pharmaceuticals, and for inactivation of dangerous pathogens to enable their safe handling. The Joint FAO/IAEA Program first promoted discussion and scientific exchange on the value of radiation attenuation to develop vaccines against animal parasites nearly 50 years ago (IAEA 1964). In a series of Expert Meetings, the subject was discussed extensively and a wide range of parasitic organisms including *Dictyocaulus* spp., *Ancylostoma*, *Echinococcus*, *Schistosoma* spp., *Fasciola* spp., *Haemonchus*, *Trypanosoma* spp., *Anaplasma*, *Babesia* spp, and *Theileria* spp. were identified as potential targets for the development of irradiated vaccines (IAEA 1968, 1970, 1973, 1981). This avenue of research was eventually put into abeyance, principally owing to the advent of molecular technologies that offered the possibility of identifying specific protective molecules that would confer resistance and could then be genetically engineered to produce a vaccine. This search for new vaccines has led to novel strategies including peptide vaccines, recombinant vector vaccines, gene-deleted vaccines, marker vaccines, DNA vaccines, synthetic vaccines, and edible vaccines. There have been some highly successful outcomes; recombinant vaccines have been developed which can prevent infection in domesticated livestock infected with *Taenia ovis*, *Taenia saginata*, *Taenia solium*, *Taenia multiceps*, and *Echinococcus granulosus. T. ovis* and *T. saginata* are economically significant parasites while *T. solium* and *E. granulosus* are zoonotic diseases and the vaccines are useful for treating animals to prevent human infections (Lightowlers 2006; Lightowlers et al. 2003; Gauci et al. 2008; Petavy et al. 2008). Other significant developments have been in viral vaccines, e.g., rabies (Lubroth et al. 2007) and in protection against *Boophilus* ticks (Canales et al. 2009). Against other pathogens, such as *Babesia* and *Theileria*, although protective antigens have been identified, effective vaccines have not materialized (Brown et al. 2006) and vaccination dependent on infection and treatment methodologies is still in use (Morrison and McKeever 2006; Di Giuolo et al. 2008). Similarly, in spite of the considerable success with cestode parasite vaccines, there has been no similar development with trematode and nematode parasites (Hein and Harrison 2005). It is likely that protective subunit vaccines will require the inclusion of multiple immunogenic proteins for them to be effective, considerably complicating the task of creating effective immunogenic antigens. One of the advantages of live, attenuated vaccines, and a reason to re-investigate the potential of radiation attenuation, is their potent immunogenicity since the organisms are still able to replicate and behave initially in a similar manner to a natural infection, thereby stimulating the immune system to secrete the immunoregulatory products and induce the cellular activation that would normally occur. Such vaccines are sometimes produced locally and are not therefore commercially available products; examples include *Eimeria* spp., *Toxoplasma*, *Babesia bovis*, *Babesia bigemina*, and *Theileria annulata* (Vercruysse et al. 2007). In fact, the most successful category of vaccines presently in use against major transboundary, infectious animal diseases such as foot and mouth disease, contagious bovine pleuropneumonia, Rift Valley fever, etc. are live vaccines, including attenuated vaccines (Lubroth et al. 2007). Indeed, one of the most successful veterinary vaccines is the attenuated tissue culture vaccine that was used in the control and eradication of rinderpest. Thus, in spite of the considerable research effort into developing recombinant vaccines, conventional vaccine technology still plays a major role in combating animal diseases (Lubroth et al. 2007).

Although only a small number of veterinary vaccines have been produced by using radiation attenuation, it is worth re-examining the potential of the process since it has been a considerable time since there has been any significant investigation. Also, a number of recent innovations in technology suggest that it could provide a viable alternative to other attenuation techniques, particularly with pathogens where recombinant vaccines have not yet delivered effective products. For instance, considerable success is being achieved in the development of an anti-malarial irradiation-attenuated sporozoite vaccine (Hoffman et al. 2010) for use in humans. In addition, new advances in the cryopreservation of somatic cells through freeze-drying offer the potential of preserving irradiation-attenuated whole-cell organisms in a way that was not previously feasible (Loi et al. 2008a, b; Natan et al. 2009).

### Radiation-attenuated vaccines for helminth parasites

Parasitism by gastrointestinal nematodes is a serious worldwide constraint on livestock productivity causing economic losses from decreased production, mortality, and the costs of continuous treatment and prevention measures. Drugs are an essential component of the management practices used in preventing clinical disease and central to the control of helminth parasites. However, since many of the drugs have been in use for many years, drug resistance is prevalent, to the extent that in the case of nematode worms every class of anthelminthic drug now in use shows signs of resistance in all livestock species (Kaplan 2004). The development of vaccines for multicellular organisms with complex genomes is a formidable task since the immune response will be multi-faceted and involve a range of mechanisms so that any vaccine will be required to activate a number of different pathways. Central to the concept of developing a vaccine is how to evaluate its efficacy and its expected impact on the occurrence of disease in the livestock population. Effective anthelminthic drugs completely clear an animal of parasites, but for a parasite vaccine this is probably not possible, nor may it even be necessary. Computer modeling has shown that for some nematode species, e.g., *Trichostrongylus*, a vaccine efficacy of 60% in 80% of the animals was sufficient to prevent disease (Barnes et al. 1995). This level of protection would ensure that pasture was not contaminated with large numbers of worm eggs, but in the case of a more prolific egg producer, such as *Haemonchus*, a vaccine efficacy of 70% would be needed to ensure that pasture contamination was minimized. Successful protection against challenge infection requires to meet three parameters: first, a reduction in the infectivity of larvae, so that establishment of adult worms is compromised, and the worm burden is lower; second, a concomitant lowering of the pathogenicity; and third, an effect on female worm fecundity so that egg numbers are decreased (Loukas et al. 2006).

Nematode parasites have been controlled for many years using anthelminthic drugs, but the progressive development of drug resistance can compromise their ability to control infection. Furthermore, even after successful treatment, individuals become susceptible to reinfection within 4–12 months. Alternative methods to chemical control include vaccination using radiation attenuation. The first irradiation-attenuated vaccine against a nematode parasite was developed for *Ancylostoma caninum* (Miller 1964, 1971, 1978). The third-stage larvae of *A. caninum* were attenuated by X-ray doses >300 Gray (Gy). Intestinal establishment of the adult worms in the intestine was reduced; few male worms survived and females were often sterile. Dogs given 1,000 irradiated larvae did not develop clinical disease. Single or double subcutaneous vaccination of larvae irradiated with 400 Gy successfully protected against challenge, achieving 90% protection after double vaccination. Approximately 75% of the irradiated larvae failed to establish in the gut, probably because they remained in the lungs where they died. The presence on the larvae in a migratory location was considered the main reason for their stimulating a high level of resistance. When normal, un-irradiated larvae were inoculated subcutaneously, they migrated to the intestine in a very short time and few were lost in the lungs during the migration. It was found that X-rays and gamma rays (from a ^60^Co source) were equally effective in delivering attenuation, but temperature and concentration of larvae were important factors to standardize, otherwise there was considerable variation in the effectiveness of irradiation attenuation. The vaccine was produced commercially in 1973, but after only 2 years it was discontinued due to low acceptance by veterinarians and poor sales. One of the reasons for the reluctance of veterinarians to use the vaccine was that vaccinated animals continued to shed eggs. For many other diseases, vaccines are often completely sterilizing thereby protecting both the individual vaccinated and unvaccinated animal thereby contributing to herd immunity. In the case of hookworm and other nematode parasites, this ideal is probably unattainable. However, the benefits would be seen in a reduction in worm burden and loss of blood from feeding worms and consequently a reduction in morbidity and mortality. It is necessary in these cases to distinguish between a hookworm infection and hookworm disease (Miller 1978). Although the radiation-attenuated vaccine failed commercially, conceptually it has continued to provide valuable insight into the mechanisms that are responsible for inducing immunity. Larvae are less motile in vaccinated animals than in animals with a primary infection, and it is suggested that this reduced motility results in a failure of larvae to evade the immune reaction (Babayan et al. 2005). Vaccinated dogs when challenged with infective larvae respond primarily to antigens of <20 kDa that are present in excretory/secretory (ES) products from adult parasites (Boag et al. 2003). In addition to a high production of antibodies against an antigen designated ASP-2, an ES product that has potential as a vaccine antigen, and strong proliferation from PMBCs to antigens of third-stage larvae, there were also marked cellular responses. Interleukin-4 production was observed in relation to IFN-γ. The antibodies induced by attenuated larvae inhibited the penetration of infective larvae through tissue, and this is a possible mechanism for the sequestration of larvae in the lungs of vaccinated dogs. It was suggested that a TH2 response is required for generation of protective immunity against hookworm, and that the ES molecule released during the establishment of the larval developmental stage are probable targets of this response (Fujiwara et al. 2006).

One of the most important blood-feeding nematode parasites is *Haemonchus contortus*, a ubiquitous parasite found in temperate regions as well as in tropical and sub-tropical regions where it has serious consequences for smallholder farmers whose livelihoods depend on the rearing of sheep and goats. Irradiation-attenuated vaccines have been shown to be of some value in protecting against infection. Adult worm-free sheep were protected against challenge with 10,000 infective third-stage larvae, after two doses of 10,000 larvae attenuated by exposure to 600 Gy from a ^60^Co source. Resistance was present within 6 days of the last vaccinating dose. IgA and IgG antibodies were present in the abomasum mucus of most sheep and appeared to be implicated in the protective immune response (Smith and Christie 1979). Further experiments confirmed a vaccine efficacy up to 86% in animals older than 6 months, but in lambs of 2 months the response was poor (Mulligan et al. 1961; Smith and Angus 1980), and this relative low efficacy in young lambs precluded the development of the vaccine further. Interestingly, it later transpired that through regular passage, the larval preparation used for attenuation became more radiosensitive, and the dose of gamma rays had to be reduced to 400 Gy from 600 Gy; otherwise, the irradiated larvae failed to induce immunity (Sivanathan et al. 1984).

There have been several other experimental studies on the value of irradiated nematode vaccines. Gould et al. (1953) showed that *Trichinella spiralis* larvae were adversely affected by X-irradiation with reproduction of trichinae largely prevented by exposing larvae *in vitro* to 35 Gy while maturation to adult forms was largely prevented by exposure to 50 or 60 Gy. In pigs, immunity to challenge with infective larvae was prevented by administration of 50,000 larvae attenuated by exposure to 100 Gy from a ^60^Co source (Cabrera and Gould 1964). *Trichostrongylus colubriformis* is another nematode worm that attaches to the lining of the abomasum of sheep where its blood sucking leads to signs of weakness, poor growth, scouring, poor reproduction, and, in severe infestations, death. A dose-dependent vaccine response is seen with irradiation-attenuated vaccines: 5,000 irradiated third-stage larvae is the threshold level, but with two doses of 20,000 larvae irradiated at 600 Gy, a 78% level of protection is provided in lambs challenged 1 month after the last immunizing inoculation. In a laboratory animal model, the gerbil, strong mucosal antibody responses were detected following vaccination and challenge (Maclean et al. 1986). *Nematodirus battus* is one of the most pathogenic organisms that infect sheep in cool temperate climates, usually arising in May and June. Infected lambs develop acute enteritis with watery diarrhea, accompanied by inappetence and weight loss, and die if not treated. Six-week-old lambs vaccinated with 20,000 gamma-irradiated L3 larvae showed a 66% reduction in worm burden when challenged with 50,000 infective larvae a month after vaccination, suggesting that further studies into the benefits of vaccination are worthwhile (Winter et al. 2000). Radiation-attenuated L3 larvae of *Strongylus vulgaris* were used to protect ponies against infection. Larvae irradiated with 70 or 100 Gy were effective in inducing protective immunity; recovery of worms was lower in vaccinated ponies and clinical sings were less obvious. Protection was associated with an anamnestic eosinophilia and recognition of *S. vulgaris* L3surface antigens (Klei et al. 1982; Monahan et al. 1994).

The most significant and successful commercial development of an irradiated vaccine was that for use against *Dictyocaulus viviparus*, the cause of “husk” in cattle (Jarrett et al. 1960). This parasite is a nematode that inhabits the bronchi and tracheae of cattle, causing a severe and sometimes fatal disease. The parasites were attenuated by irradiation at doses of 200 to 600 Gy to third-stage larvae. The lowest dose was insufficient to alter the infectivity of the larvae, and doses of 600 Gy damaged the larvae so that they did not induce protective immunity; the larvae are completely inactivated and fail to migrate to a location where they can initiate an effective immune response. An intermediate dose of 400 Gy attenuated infectivity sufficiently so that the larvae were unable to develop into adult worms but could still induce protective immune responses in cattle under both experimental and natural conditions (Jarrett et al. 1960). Although X-rays were used in the initial studies, γ-irradiation was employed subsequently. Two oral doses of 1,000 irradiated larvae are effective in reducing worm burdens by up to 95%, protecting against clinical disease and providing protection for up to 12 months. The immunity is not a sterile one, but in areas where the disease is endemic, natural infections provide a boost that can result in a life-long immunity. Animals vaccinated with the radiation-attenuated larvae elicit a strong antibody response against immunodominant N-glycan moieties (Fujiwara et al. 2006). Being able to keep vaccines in conditions where long-term storage and distribution are easily achieved is a critical factor in the use of vaccination for animal disease control; unfortunately, cryopreservation of the irradiated *Dictyocaulus* reduces viability by 70% (James and Peacock 1986) and consequently there is no long-term storage option for the vaccine and it has to be stored at <8°C, under which conditions it has a shelf-life of approximately 3 months. Nevertheless, in spite of these apparent drawbacks, the vaccine became a mainstay of control of husk for over 50 years.

Parasitic bronchitis of sheep and goats also occurs in hilly regions of India and neighboring countries. Several species of strongyloid nematodes are involved but the most prevalent and most pathogenic is *Dictyocaulus filaria* which causes high mortality and morbidity in young animals. Immunity can be induced by vaccination of lambs with two doses of 1,000 larvae irradiated at 400 Gy by X-rays or gamma rays (Sokolic et al. 1963). When vaccinated animals were challenged a lower number of larvae and reproductive female worms were recovered from them, the clinical and pathological effects of infection were less pronounced, parasite development, particularly oogenesis, was lower, and there was no mortality in vaccinated lambs compared with unvaccinated controls. Effective control of this parasite in India was made possible using a gamma-attenuated *D. filaria* vaccine developed in 1971 to treat 6-week-old lambs as part of sheep husbandry practice. In another study, sheep vaccinated with 500 Gy irradiated third-stage larvae on two occasions a month apart and then allowed to feed in natural pasture showed a high degree of resistance against *D. filaria* and were also protected to some degree against infection with other nematodes (Dhar and Sharma 1981). Over an 8-month observation period, there was a reduced prevalence of infection and significantly lower output of larvae in the feces of vaccinated lambs.

Trematode worms can be divided into three types depending on where the adults establish in the host, hence, blood flukes, liver flukes, and rumen and intestinal flukes. There are several different species of blood flukes, but the most important are *Schistosoma japonicum* and *Schistosoma bovis*. The former is a zoonotic species that occurs in cattle, buffalo, sheep, goats, and pigs in China and South East Asia and is a serious disease threat to millions of people in Asia. *S. bovis* is a parasite of cattle, sheep, and goats in Africa where it causes significant mortality. Liver fluke infections occur worldwide, the most important species being *Fasciola hepatica* and *Fasciola gigantica*, and they pose a threat in many developing countries. They can infect cattle, sheep, goats, horses, and pigs, and there are cases of zoonotic infections too. In Europe and America, *F. hepatica* is the only species that occurs, but in Africa and Asia both species occur, often overlapping in distribution. The third group of trematode parasites, represented by the paramphistomes, is of less economic importance than the blood and hepatic flukes.

Significant resistance to natural infections with *F. hepatica* was obtained in calves inoculated with metacercariae attenuated using 30 Gy γ-irradiation. The mean fluke burden of vaccinated calves was 61% less than that of the control, unvaccinated cattle (Nansen 1975). The resistance was reflected not only in reduced parasite burdens and low fecal egg counts but also in the stability of glutamyltranspeptidase levels in the vaccinated calves compared with non-vaccinated controls. A small proportion of the irradiated larvae were still able to reach and mature in the bile ducts. Conversely, attempts to induce immunity in sheep using various numbers of larvae and irradiation levels and different vaccination schedules failed. Vaccination of sheep with either 100 or 1,000 irradiated (25 Gy) metacercariae on two occasions 6 weeks apart did not induce immunity against challenge with *F. hepatica* 6 weeks after the second vaccinating dose as measured by recovery of flukes from liver and bile ducts. There was no reduction in the pathogenic effects of challenge infection as measured by reduced packed cell volumes and weight gain (Campbell et al. 1978). In other experiments, sheep were vaccinated with two doses of 2,000 metacercariae 4 weeks apart, irradiated at 70, 100, or 150 Gy. Although parasite viability was compromised and strong humoral antibody responses were generated, there was no protection (Creaney et al. 1995a, b). The mechanisms of action of irradiation attenuation with 10 or 40 Gy on the juvenile stages of *F. hepatica* seem to be directed at expression of cathepsin-B protease and wheat germ agglutinin (WGA)-specific sugars and concanavalin A (ConA)-specific sugars which may be detrimental to parasite invasion and contribute to the immune responses (Creaney et al. 1995a, b). In lambs vaccinated with *F. gigantica* metacercariae irradiated at 30 Gy, there was an 80% reduction in worm burden and fewer signs of hepatic damage than unvaccinated controls (Agadira et al. 1987). In zebu calves vaccinated with 30 Gy irradiated metacercariae, there was a 77% reduction in worm burden when the calves were challenged with 1,000 normal cercariae; in animals vaccinated with 200 Gy irradiated parasites, there was a high level of resistance shown by an 88% reduction in worm burden and mild effects on hepatic function (Younis et al. 1986). In goats, 400 metacercariae of *F. gigantica* irradiated at 30 Gy stimulated resistance to challenge infection 8 weeks after vaccination (El Sanhouri et al. 1987).

*S. bovis* is an economically important parasite of cattle and other domestic livestock in Sudan where control by chemotherapy and molluscicides is impractical. Sheep were vaccinated with 10,000 or 20,000 schistosomula irradiated at 60 Gy and then challenged with 6,700 live schistosomula. In sheep vaccinated with 10,000 organisms, there was a 71% reduction in the mean worm burden compared with the controls and the mean densities of eggs in the tissues were reduced by 75–82% (Taylor et al. 1979). Immunization with 20,000 irradiated schistosomula did not increase the efficacy of vaccination. Both groups of vaccinated sheep had much milder lesions in the intestines than control individuals. There was also a clear reduction in the severity of liver lesions in the sheep given the lower dose of irradiated schistosomula. In the case of the related parasite of sheep, *Schistosoma mattheei*, from southern Africa, four doses of 10^4^ cercariae irradiated at 6 Kr that were given by percutaneous infection at 4-week intervals induced a 74% reduction in infection compared to control (Taylor et al. 1976). No significant differences were seen in histopathology of the liver of the sheep. In another experiment, four doses of cercariae irradiated with 60 Gy were used to vaccinate sheep by percutaneous infection; skin-transformed schistosomula were administered by intramuscular injection and by intravenous injection and syringe-transformed schistosomula were administered by intramuscular injection. The reduction in worm burden achieved by these different procedures was respectively 72%, 61%, 77%, 56%, and 78%. These results show that effective immunization is not dependent on the presence of a mature worm infection or on cercarial penetration of the skin by the immunizing infection. Zebu cattle were vaccinated by intramuscular or subcutaneous inoculation with 10,000 schistosomula irradiated at 30 Gy. They were then challenged with 10,000 normal schistomula. Vaccinated animals had significantly lower egg and adult worm counts, milder pathological effects, and higher growth rates and body composition than control animals (Bushara et al. 1978). In field trials in endemic areas of Sudan, a single i.m. injection of *S. bovis* cercariae irradiated with 30 Gy reduced worm burdens by 60% and egg production by 80% (Majid et al. 1980; Aradaib and Osburn 1995). Similar results were obtained with sheep. Irradiated, cryopreserved *S. bovis* schistosomula were successfully used to protect sheep against challenge, and with more efficient cryopreservation techniques it should be possible to increase the efficacy of a vaccine stabilate (James et al. 1985).

The prevalence of *S. japonicum* in buffalos and cattle in China can be as high as 45%, and since bovines are a reservoir of human disease caused by *S. japonicum*, its control will benefit both livestock production and human health. Several studies using attenuated parasite larvae have given promising results (Li Hsu et al. 1984a, b). Cattle were immunized intradermally and intramuscularly with 10,000 schistosomula that had been irradiated with 240, 360, or 480 Gy. One to three immunizing doses were given and the cattle were challenged with 500 normal cercariae 30 days after the last immunizing injection. There were significantly lower numbers of adult worms in the animals vaccinated with schistosomula exposed to 32 Gy than those with 24 or 48 Gy. The reduction was greatest, 87%, in animals that were given three immunizing doses. Field trials of the vaccine were also conducted in two areas in Anhui Province; one area with a snail infection rate of 0.27% was considered lightly endemic and the other, with an infection rate in the snails of 4–8%, was considered heavily endemic for *S. japonicum* (Li Hsu et al. 1984a, b). Schistosomula were irradiated at 38 Gy and buffalo were vaccinated twice and transported to the lightly endemic area. The vaccinated buffalo showed a body weight gain of 34% compared with 21% in control, unvaccinated buffalo and 150 days after placing in the pasture the average reduction in worm counts was 75%, while egg counts were reduced by 80%. Cattle and buffalo given three doses of irradiated vaccine were placed in the area that was highly endemic for *S. bovis.* All animals lost weight, although this was slightly higher in the unvaccinated animals. However, worm burdens were reduced by 65% and 75% in vaccinated cattle and buffalo, respectively. The reduction in egg counts was 55% in vaccinated cattle and 70% in the vaccinated buffalo. Pigs vaccinated with schistosomula inactivated by 200 Gy irradiation were 95% resistant to challenge judged by fecal egg counts and pathology. Interestingly, the vaccinated pigs did not produce antibodies against a 62-kDa myosin protein, Sj62, which was thought to be implicated in development of resistance to schistosome infection (Bickle et al. 2001). The actual mechanisms of action of radiation attenuation in schistosomes are not well understood, but it is believed that for the process to be effective, attenuation must be sufficient to retard migration of larvae through the lungs. This increase in immunogenicity may be related to alterations observed on gene expression in irradiated larvae (Dillon et al. 2008).

### Radiation-attenuated vaccines for protozoan parasites

Infections with animal trypanosomoses threaten an estimated 50 million head of cattle and in regions of sub-Saharan Africa where the tsetse fly vectors of the diseases occur. Every year, African Animal Trypanosomoses (AAT) caused by *Trypanosoma vivax*, *Trypanosoma congolense*, and *Trypanosoma brucei* cause some 3 million deaths in cattle. AAT has a severe impact on agriculture in sub-Saharan Africa; for the tsetse-infested regions, overall losses, in terms of agricultural gross domestic product, have been put at US$ 4.75 billion per year. Currently, drugs are pivotal to the control of trypanosomosis, but the small number of available compounds, together with frequent reports of drug resistance to all species of animal trypanosomoses, makes it imperative that measures are taken that enable more effective use of the available drugs or, alternatively, seek alternative methods of control. Vaccination is an attractive prospect, but so far none of the various strategies devised have been successful and have not been taken beyond the experimental stage (Magez et al. 2010). These attempts have comprised inducing protective immune responses in the mammalian host, mitigating the pathogenic effects of infection, compromising transmission of infection by stopping the development of trypanosomes in the vector or by blocking the establishment of infection in the mammalian host. One of the main factors that make vaccination difficult is the existence of several different trypanosome species and antigenic variation of the parasite that provides for large variable antigenic repertoire in pathogenic trypanosomes. Given the success of γ-irradiation attenuation with a number of other helminths, viruses, and bacteria, work with trypanosomes have met with only limited success, in marked contrast to recent studies where irradiated organisms have behaved in a manner that enables them to be more effective immunogens that can sometimes elicit substantial cross-protection. One of the most important attributes seems to be in retarding development of the infective stage of the parasite to enable the induction of immunity. It is possible that successful protection requires that immune effector cells that would be stimulated in a natural infection should be preferentially primed, requiring vaccination at the skin, mucosal surfaces etc., where infection normally occurs. In the case of trypanosomes, the infection in the host is effected by the bites of tsetse flies or hematophagous biting flies after which trypanosomes develop in the skin and draining lymph nodes (Mwangi et al. 1990). However, in all experiments carried out using irradiated trypanosomes, vaccination and challenge was carried out by the intraperitoneal, intravenous, or subcutaneous routes. Between 50% and 80% of mice vaccinated with *T. congolense* that had been γ-irradiated with 600 Gy were immune to challenge after up to six immunizing injections with 2 × 10^6^ to 2 × 10^7^ inactivated trypanosomes and challenge 7 days after the last dose; dogs and cattle were not protected although the prepatent period after challenge was lengthened (Duxbury et al. 1972b). In a similar study using γ-irradiated *Trypanosoma rhodesiense*, five out of six monkeys were immune to challenge 7 days after receiving six weekly immunizing inoculations (Duxbury et al. 1972a, b). Interestingly, it was found that irradiation attenuated *T. rhodesiense* that had been cryopreserved in liquid nitrogen retained the ability to induce immunity. (Duxbury et al. 1970). Experiments with *T. brucei* in rats vaccinated with trypanosomes attenuated with 420 Gy showed an increased survival time, with some exhibiting complete immunity after three or more injections with irradiated parasites (James et al. 1973). In experiments with cattle (Morrison et al. 1982), a single dose of 10^7^ to 10^9^ *T. brucei* irradiated with 600 Gy completely protected against challenge 14 days later. High levels of protective antibodies were produced, especially in cattle in which responses were induced by intravenous inoculation. The success of the single vaccinating dose was probably due to the use of a homogeneous cloned population of trypanosomes, whereas in previous studies mixed populations of parasites were used so that the presence of several antigen types in small numbers was not sufficient to induce immunity. There has been only one attempt using irradiated trypanosomes to protect against infective metacyclic forms produced in the tsetse fly. Mice immunized three times with 1 × 10^6^ irradiated *T. rhodesiense* expressing metacyclic antigen types were immune to challenge 2 weeks after the last immunizing dose (Esser et al. 1982).

The Theileriae comprise a genus of tick-transmitted protozoan parasites infecting wild and domestic animals in many parts of the world. In East and Central Africa, the most important species *Theileria parva* represents a major constraint on the development of beef and dairy production. *T. annulata* is more widespread, causing disease in cattle in the Mediterranean region, the Middle East, India, Southern Russia, and other countries in Asia. The parasites have a complex life cycle in the mammalian host and in the arthropod vector, various species of tick. Theileriosis is controlled by dipping or spraying cattle with acaricides to kill the tick vectors. In addition, *T. annulata* can be controlled using a tissue culture-attenuated vaccine, and for *T. parva* there are a number of curative drugs and also vaccination by infection from stabilated infective particles (IPs) followed by treatment with long-acting tetracycline drugs. There have also been a number of trials of γ-irradiated vaccines to investigate the possibility of immunoprophylaxis by this method. Calves were inoculated with ground-up suspensions of ticks infected with *T. annulata* that had been irradiated at 50, 100, and 150 Gy and the response in the calves measured. The lowest dose did not affect the parasite, but at 100 and 150 Gy the parasitosis was lower in the vaccinated animals than in control animals inoculated with unirradiated parasites and the clinical signs were of reduced intensity. It was suggested that irradiation altered virulence of the parasite rather than impair infectivity. Vaccinated animals were fully resistant to challenge infection 45 days after immunization (Samantaray et al. 1980). Infective particles of *T. parva* were harvested from the tick vector, and cattle were inoculated with aliquots of suspensions of IPs irradiated at doses of 40–1,376 Gy. Doses of irradiation in excess of 80 Gy appeared to destroy the parasite. In other experiments, cattle were inoculated with aliquots of suspensions containing low and high concentrations of IPs irradiated at doses of 40–320 Gy. With low concentrations of IPs, doses of irradiation in excess of 100 Gy appeared to destroy the parasite. With high concentrations, one animal became infected when inoculated with an aliquot of a suspension irradiated at 160 Gy. In all experiments, it appeared that increasing doses of irradiation destroyed increasing numbers of IPs (Purnell et al. 1974). It was concluded that γ-irradiation was unlikely to be useful for producing a vaccine antigen against ECF is unlikely to be achieved by these means.

Another important arthropod-borne disease of cattle is babesiosis, a parasitic infection that causes significant morbidity and mortality in livestock worldwide. The most abundant species, *B. bovis* and *B. bigemina*, occur throughout most tropical and sub-tropical regions where economic losses from infection are considerable, particularly in developing countries. *Babesia divergens* is economically important in Europe. Babesiosis can be treated with anti-parasitic drugs or controlled with vaccination—a number of live, attenuated vaccines derived from the blood of infected donor animals or from *in vitro* culture are available for *B. bovis*, *B. bigemina*, and *B. divergens*. While effective, there are safety concerns and quality control demands to ensure that vaccines retain their integrity during preparation techniques that require passage of the parasites *in vitro* and in splenectomized calves. Alternative methods of attenuation utilizing γ-irradiation have proven effective experimentally, and could possibly reduce some of the problems of the current vaccines, but they have not been adopted for large-scale use. A field trial of a blood-derived *B. divergens* vaccine irradiated at 240 or 320 Gy produced an effective immune response without pathogenic effects. Vaccinated calves were protected against a field challenge that produced severe disease in unvaccinated animals (Purnell et al. 1979). A larger trial using infected blood irradiated at 280 Gy confirmed these initial findings (Purnell et al. 1981). The route of inoculation also had an effect on response to challenge: animals inoculated subcutaneously showed fewer effects than by intravenous inoculation (Taylor et al. 1983). Bovine blood and fetal calf serum are often used in cell culture techniques, and it is common knowledge that these products can be contaminated with viruses, one of the most ubiquitous being bovine viral diarrhea virus. Hence, there is a risk that if used in production of vaccines, the recipient animals could become infected. Gamma irradiation of serum inactivates the viruses of foot and mouth disease, vesicular stomatitis, rinderpest, peste des petits ruminants, Rift Valley fever, and bluetongue while maintaining growth promotion potential. Attenuated vaccines for *B. bovis* and *B. bigemina* (Rojas et al. 2006) require regular supplies of bovine red blood cells (RBCs) and serum for their manufacture, and there is a constant problem with contaminating viruses and bacteria. Irradiation of serum with 25,000 Gy has therefore been used to inactivate viral contaminants and ensure its safety and efficacy in use.

Observations on the effects of γ-irradiation on *B. bovis* showed that the numbers of parasites capable of multiplying in the host reduced as the doses increased with complete inhibition of multiplication at 500–700 Gy. At 200–500 Gy, parasites were able to multiply at a reduced rate and produced mild infections that provided a strong immunity to challenge infection. At the highest radiation doses, where no multiplication occurred, calves were not protected (Wright et al. 1980). Cattle vaccinated with the irradiation-attenuated parasites retained their immunity to challenge with a heterologous strain of *B. bovis* for over a year. When infected blood containing 1 × 10^8^ infected red cells was irradiated at 350 Gy, the infective dose was reduced to 2.5 × 10^3^ parasites, but the rate of multiplication was similar to unirradiated parasites, and the same level of parasitemia was reached in the vaccinated animals. However, the disease was not as severe. It was concluded that irradiation had produced a predominantly avirulent parasite population (Wright et al. 1980). With *B. bigemina*, inoculation of 1 × 10^10^ parasitized red cells irradiated at 240 or 360 Gy into calves produced infection, but animals inoculated with parasites treated with 480 or 600 Gy did not develop infections and they were able to survive challenge with unirradiated parasites that killed control, unvaccinated calves. The irradiated parasites could be cryopreserved without loss of immunizing properties (Bishop and Adams 1974).

Coccidiosis caused by intracellular parasites of the genus *Eimeria* is a major enteric disease of intensively reared poultry, causing production losses of more than $800 billion worldwide (Williams 1999). The disease can be controlled by anti-coccidial drugs and vaccines composed of live and attenuated parasites, and studies on the use of recombinant vaccines are also in progress (Dalloul and Lillehoj 2006). Oocysts of *Eimeria tenella* and *Eimeria maxima* exposed to 200 Gy induced a protective immune response in chickens; irradiated sporozoites invaded host epithelial cells but showed only minimal development and by 72 h had disappeared. Vaccinated chickens showed no weight loss when challenged with live oocysts, but did develop lesions in the intestine (Jenkins et al. 1991, 1993).

*Neospora caninum* is a protozoan parasite that causes neuromuscular paralysis in dogs and abortions in cattle. *N. caninum* has considerable impact upon beef and dairy industries worldwide (Reichel and Ellis 2006). The important host immune responses to *N. caninum* include CD4^+^ T cells, the Th1 cytokines IL-12, interferon gamma, and IgG2a isotype antibodies. In a murine model of the disease, mice could be protected from lethal challenge infection with 2 × 10^7^ live tachyzoites using two intraperitoneal injections at 4-week intervals with 10^6^ tachyzoites irradiated with 528 Gy. Splenocytes from mice collected 5 and 10 weeks after vaccination secreted significant levels of interferon g, interleukin (IL)-10, and some IL-4. IgG1 and IgG2a isotype antibodies were present in the serum of infected mice (Ramamoorthy et al. 2006). To determine the efficacy of the vaccine in preventing vertical transmission of *N. caninum*, mice were vaccinated and bred after administration of a booster dose 4 weeks after the primary vaccination. Antigen-specific IgG, IFN-γ, and IL-10 were detected in the vaccinated pregnant mice. Vaccinated mice were then challenged with 5 × 10^6^ *N. caninum* tachyzoites between days 11 and 13 of pregnancy. Protection against vertical transmission of *N. caninum* was 34%. Brain tissue of the fetuses showed that there were highly significant differences in parasite burdens between control and vaccinated animals (Ramamoorthy et al. 2007).

### Radiation-attenuated microbial pathogens

The basic tenets of irradiation attenuation, namely that inactivated microbial pathogens retain their immunogenicity and induce protective immune responses in the host, were established during experimental studies on *Rickettsia tsutsugamushi* (Eisenberg and Osterman 1978). It was found that immunogenicity was little compromised between irradiation does of 2,000 and 4,000 Gy. Mice vaccinated using an intermediate dose of 3,000 Gy was completely protected against homologous challenge. In contrast, mice vaccinated with a formalinized vaccine were not protected. Specific cellular immune responses were detected in mice given the irradiated vaccine, but could not be demonstrated in mice given the chemically treated rickettsiae. It was concluded that irradiated rickettsiae share similar antigenic properties to irradiated organisms, thereby enabling the stimulation of protective immunity. The immunogenic properties of irradiated vaccines, particularly in relation to enabling retention of metabolic activity and the adjuvant and antigenic properties of the organisms, were confirmed by Datta et al. (2006). Intraperitoneal injection of *Listeria monocytogenes* irradiated at 6,000 Gy induced protective immune responses in mice that were accompanied by extensive protective CD4^+^ and CD8^+^ T-cell memory responses. Moreover, lyophilized, irradiated *Listeria* organisms were also able to protect mice, with implications for the safe and easy storage and transport of such vaccines (Datta et al. 2006). The benefits of using as vaccine organisms that had been inactivated, but metabolically active, have also been established with *Brucella* (Magnani et al. 2009). Intraperitoneal injection with chemical or heat-treated organisms provided only limited vaccine efficacy, but *Brucella* irradiated at 3,000 Gy while preventing replicative activity retained the properties of live bacteria remaining metabolic activity, transcriptional activity, ability to establish in macrophages, induced cytotoxic T cells, and protected against lethal challenge infection. This characteristic of irradiation-attenuated vaccines might contribute to induction of more comprehensive immunity. For example, mice vaccinated with *B. neotomae* attenuated with 2,500–3,000 Gy gamma rays protected against infection with heterologous, virulent organisms of *Brucella melitensis*, *Brucella abortus*, and *Brucella suis* (Moustafa et al. 2011). There is also evidence that gamma-irradiated *Bacillus anthracis* spores induce mucosal immunity in guinea pigs when administered *per os. B. anthracis* spores irradiated with 20,000 Gy induced specific IgG1 and IgA antibody responses against a recombinant protective antigen (Aloni-Grinstein et al. 2005). Vaccination against *Pasterurella* can also be induced in mice using bacteria irradiated with 6,000 Gy. In contrast, with *Burkholderia mallei*, the causative organism of glanders in equines, although organisms irradiated at 21,000 Gy were able to induce T-helper cell cytokine responses in vaccinated mice, there was no evidence of protective immunity (Amemiya et al. 2002). Results with *Leptospira icterohemorrhagiae* were also equivocal, possibly due to differences in irradiation dosage. Guinea pigs were successfully protected against infection by vaccination with leptospira irradiated at 500 Gy (Hubbert and Miller 1965), but when the inactivation dose was increased to 2,000 Gy, there was no protective effect (Babudieriq et al. 1973). Bovine anaplasmosis is caused by a tick-borne rickettsia, *Anaplasma marginale*. Cattle vaccinated with infected red cells γ-irradiated with 700–900 Gy showed marked and prolonged cell-mediated and humoral immune responses and had lower parasitemias and anemia than in control animals (Sharma and Bansal 1986).

Irradiation is a very reliable procedure for inactivating viruses, as it does affect viral proteins and structure and also shows promise for formulation of cross-protective vaccines, thereby reducing the numbers of vaccines that might otherwise be required (Alsharifi et al. 2009). Gamma irradiation was effective in inactivating bluetongue virus without compromising the presentation of antigenic determinants that stimulated production of neutralizing antibodies in both mice and sheep. Virus preparations from brain and cell cultures were inactivated with 60,000 to 100,000 Gy from a ^60^Co source. The vaccine induced a cell-mediated immune response in mice and sheep were protected against infection (Campbell 1985; Campbell et al. 1985). The dose of irradiation did not appear to affect the immunogenicity of the virus. Foot and mouth disease (FMD) is inactivated by irradiation within the range 40,000–44,000 Gy (Motamedi-Sedeh et al. 2009). In guinea pigs, irradiation-attenuated FMD viruses induced neutralizing antibodies that persisted for up to 8 months, post-vaccination—a similar duration to that obtained using conventional FMD vaccine. Venezuelan equine encephalitis virus (VEEV) is a mosquito-borne and aerosol-transmitted infection of horses (in which the virus multiplies) that presents an emerging zoonotic disease threat to humans, and is in addition a potential biological weapon. The disease is endemic to many areas of Central and South America. In horses, the disease manifests either as a mild fever that progresses into severe and often fatal encephalomyelitis, although it can produce an acute disease causing sudden death; mortality can be as high as 80%. Humans are infected by mosquitoes and the disease produces flu-like symptoms that result in death in some instances. Aliquots of VEEV at 3 × 10^5^–3 × 10^7^ pfu/ml were irradiated with 400–50,000 Gy and then tested for infectivity; irradiation of at least 50,000 Gy was required to abolish infectivity (Fine et al. 2010). There was also a 32–50% reduction in epitope binding activity concomitant with loss of infectivity. An attenuated vaccine prepared from VEE viruses irradiated with 50,000 Gy was effective in inducing protection in mice against subcutaneous challenge with live virus, although immunity was not necessarily associated with high levels of neutralizing antibodies (Martin et al. 2010); however, the vaccine was less effective in protecting against aerosol infections. It was not necessary to include adjuvants in the formulation to ensure induction of immunity.

Chemically inactivated influenza virus vaccines rely on protective antibody responses and do not elicit the cytotoxic T-cell responses that are important in recovery from primary infections. Influenza A virus irradiated with 120,000 Gy induced T-cell responses in mice and protected them against lethal challenge with a heterologous strain of virus (Müllbacher et al. 1988). Mice given a single intranasal vaccination with influenza A virus, inactivated by irradiation at 10,000 Gy, were protected against homologous and heterologous challenge with influenza A viruses, including H5N1 (Alsharifi et al. 2009). Cross-protective immunity was mediated mainly by T-cell responses and γ-irradiated viruses did not generate neutralizing antibodies or cross-protective antibodies (Furuya et al. 2010). The provision of vaccines for influenza is particularly problematic, given the ability of the viruses to mutate, potentially acquiring the ability to translate into new pandemic strains with capacity to cross the species barrier, with serious consequences for both animals and humans (Alsharifi and Müllbacher 2010). The recent epidemics caused by influenza A, H1N1 (so-called swine flu), and H5N1 (Highly Pathogenic Avian Influenza) serve to illustrate the problems faced by the authorities in providing suitable vaccines to meet the challenge posed by such disease outbreaks. Irradiated vaccines, with their ability to promote both cellular and humoral immunity, and the ability to cross-protect could become potent alternatives to present-day vaccines (Alsharifi and Müllbacher 2010).

### Radiation attenuation applications for use in diagnostic tests

It is often necessary to transport specimens required for diagnosis across international borders, and this raises issues related to biological security for both animals and man. Legislation may require that sera are treated to inactivate any pathogens that are present in specimens. Several techniques can be employed, including chemical sterilization or heat treatment, but these might have deleterious effects on samples, compromising test sensitivity and specificity. As noted above, gamma irradiation represents an effective method for virus inactivation, but tests may require adjustment, as irradiation can in some circumstances affect their diagnostic sensitivity and specificity. Sera from cattle infected with *T. congolense*, *T. vivax*, and *T. brucei* were irradiated with 30,000 Gy and then used in enzyme-linked immunosorbent assays (ELISAs) for detection of invariant trypanosomal antigens (Rebeski et al. 1998). In samples where there were unequivocal positive or negative ELISA results, irradiation had no effect on the diagnostic sensitivity of the assays—all samples shown to be positive before irradiation remained positive and those shown to be negative remained negative. However, a statistically significant reduction in signal in each of the ELISAs was observed using the irradiated sera. Serum samples where the values for optical density before irradiation were close to the diagnostic cut-off showed a negative bias after irradiation. Without correction, these samples gave more false-negative test results upon irradiation. It was therefore necessary to adjust the diagnostic negative/positive threshold of the ELISAs using defined irradiated serum samples; otherwise, the frequency of false-negative results was increased. When irradiated sera were used in indirect ELISA tests for detection of antibodies to *T. congolense* and *T. vivax* (Rebeski et al. 2001), optical density values were higher for most gamma-irradiated antibody positive and negative test samples than in the unirradiated samples. The intraplate precision and agreement between tested and expected values of measurements, however, were not altered. Although there was a bias of higher measurement values after gamma irradiation, this could be compensated after readjustment of the cut-off points to obtain best separation of antibody-positive and negative samples. Irradiation is also useful in inactivating samples that contain pathogens harmful to laboratory personnel. *B. anthracis* samples exposed to 25,000 Gy could still be detected by real-time PCR and in fact detection of DNA targets was enhanced; in ELISA tests, the direct ELISA was affected negatively by irradiation, but a sandwich ELISA was unaffected (Dauphin et al. 2008). Irradiated antigens have also been used in the diagnosis of Rift Valley fever (RVF), where classical detection methods pose significant health risks and require as long as 7 days to produce results. A sandwich ELISA to detect IgM and IgG antibodies to RVF virus in domesticated livestock had high diagnostic sensitivity (Paweska et al. 2003b), while an indirect ELISA had similar high diagnostic sensitivity and specificity in livestock and in a number of different wildlife species (Paweska et al. 2003a). Irradiation of rinderpest virus with doses of 10,000–60,000 Gy led to a dose-dependent reduction in activity as measured by virus neutralization, ELISA, and IFAT. In a competitive ELISA, bluetongue virus (BTV) antigen irradiated at 10,000–60,000 Gy also showed a loss in immunoreactivity at the higher dose levels (Afshar et al. 1991). A gamma-irradiated inactivated cell culture derived African horse sickness viral antigen has been used in a blocking ELISA (B-ELISA) for detecting antibody to a subgroup-reactive epitope of African horse sickness virus (House et al. 1996). Foot and mouth disease virus irradiated from 10,000 to 45,000 Gy appeared to have unaltered antigenicity in blocking ELISA tests (Motamedi-Sedeh et al. 2009).

### Nuclear technologies in the diagnosis of infectious diseases

Radioimmunoassays (RIA) were introduced some 50 years ago, heralding a technology that enabled the measurement of minute amounts of analytes in various biological systems with specificity, sensitivity, and precision that until then was impossible to achieve. Radioactive tracers are suitable for labeling molecules because their behavior in biological reactions is identical with that of non-isotopically labeled proteins. Several different isotopes have been used including ^14^C, ^3^H, ^125^I, ^35^S, and ^32^P although most proteins, peptides, and glycoproteins are labeled with ^125^I. Radiolabels were instrumental in delivering the means by which antigens of disease pathogens (viruses, bacteria, parasites) could be identified in various host tissues and secretions by specific DNA hybridization, allowing differentiation between active and previous infections and on a more fundamental level, the potential for phylogenetic characterization and epidemiological studies.

The term immunoassay covers the wide range of qualitative and quantitative processes involved in antigen/antibody interaction, both the binding of antigen with an antibody from which the concentration of the antigen is determined and also methods in which the aim is to detect the presence of antibody in a system. The tests that heralded the introduction of the first generation of immunoassays were isotopically based competitive methods using radiolabeled antigen; an unlabeled antigen being measured competed with known amounts of the same antigen labeled with ^132^I for binding sites on a limited quantity of high affinity antibody. Initially, these assays were used to measure insulin levels in blood, but the combination of high specificity and sensitivity of RIAs led to their widespread application not only in the field of endocrinology but also in many other fields encompassing human and veterinary medicine, environmental sciences, and the food industry, enabling the testing and detection of thousands of substances of biological importance. The use of highly sensitive DNA probes for diagnosis offered an opportunity to detect organisms that were often difficult to detect by conventional techniques and also provide specific identification since the probes were complementary sequences to the organism’s own DNA. For labeling nucleic acids, ^32^P is favored because of its high energy and thus the need for only short counting times, or shorter exposure for producing autoradiographs. For disease diagnosis, RIAs were modified so that they could be used in plastic tubes or polyvinyl microtiter plates, thereby making the technology simpler. The use of plastic supports made possible further refinements and modifications that would ultimately lead to the replacement of assays relying on radionuclides with ones based on enzymes. Among the reasons for this were the potential disadvantages of isotopes due to their hazardous nature associated with radioactivity (although alternatives are not without risk viz. carcinogenic chemicals), cost of equipment, shelf-life of radiolabeled reagents, and increasing health and safety issues that required non-isotopic methods to be used if they were available. Additionally, with the demand for ultra-sensitive methods, enzyme labels, with their capacity for catalyzing reactions, were considered to be highly applicable. In addition, the non-radioactive tests could more easily be used in non-specialist laboratories and for development of penside tests.

Enzyme immunoassays, in the format of antigen- or antibody-coated microtiter plates, sometimes using monoclonal antibodies and recombinant antigens, became a mainstay for animal disease diagnosis. APU pioneered the development of assays to diagnose a number of infectious and parasitic diseases and create the infrastructural basis to enable MS to provide diagnostic processes that were reliable, accurate, and quality controlled. This has been a major contributory factor in the successful deployment of tests for a number of major livestock diseases including foot and mouth disease, brucellosis, rinderpest, and animal trypanosomiases, and their use in major control or eradication campaigns.

Serological diagnostic tests, however, even quantitatively highly sensitive immunoassays, still have a number of drawbacks. First, there is a lag phase from the time of infection to appearance of antibodies; second, it may not be possible to differentiate between vaccinated and infected animals; third, their diagnostic sensitivity and specificity may be sufficient to use as screening tests, but unacceptable in situations where it is necessary to confirm that livestock are free from infection following control and eradication. Monoclonal antibody-based tests can be used for direct detection of antigens but are not suitable in all situations.

This need stimulated the creation of another wave of isotope-based technologies relying on nucleic acid hybridization to detect genetic material in host tissues. This allows direct identification of infected animals as well as, by selecting appropriate nucleic acid sequences, providing information that may be of epidemiological importance in relation to the strain type or variant of the agent. These tests depend on the preparation of suitable probes to a target DNA that is then labeled using tracers such as ^32^S or ^32^P so that hybridization to the target can be detected. Radioactive labeled probes were used in dot blot hybridization assays where denatured test samples were applied to nylon membranes. These membranes were then heat treated and a pre-hybridization step introduced before the stage of hybridization. This was carried out by incubating the membranes with the labeled DNA probe under varying buffer conditions and time and temperature conditions. After extensive washing to remove unbound reactants, the hybrids are then detected by autoradiography. These early techniques were not sufficiently sensitive to detect very low concentrations of the target molecule in clinical samples—as is the situation in infectious diseases. To circumvent this problem, *in vitro* methods of nucleic acid amplification were introduced, utilizing heat-resistant nucleic acid processing enzymes in the polymerase chain reaction (PCR), where, by applying a number of cycles of amplification, there is an exponential increase in the amount of the specific target. The synthesis is carried out repeatedly in a thermal cycler to replicate the DNA. Although this technique increased the sensitivity of the tests and speeded up the hybridization stage, the detection of amplicons by autoradiography was a slow process; although exposure of film for 4–24 h was sometimes sufficient to reveal DNA, the most sensitive detection required long exposure (up to 7 days) and very low temperatures (−70°C). Obviously, the aim of diagnostics is to deliver tests that are easy to perform and for which the results are rapidly and easily interpreted; clearly, PCR in this form would not provide for rapid diagnosis, and in addition, such procedures would be difficult to transfer to laboratories in developing countries. Hence, as was the case with immunoassays, technologies that no longer relied on radioisotopes were adopted for molecular characterization. These alternative procedures use chemicals including ethidium bromide or SYBR Green that can be detected rapidly by fluorescent light, thereby speeding up and simplifying interpretation of results; hence, PCR techniques are now widely used for disease diagnosis.

### Radiolabeled probes and radioimmunoassays for the diagnosis and characterization of parasitic infections

Many routine diagnostic tests provide an indirect identification of the parasite by the detection of antibodies in the serum of the infected animal. Serology involving the detection of antibodies does not allow easy differentiation between recent or latent infections, whether an animal is a carrier, or if it is free from infection following successful drug treatment. Nucleic acid-based diagnostics potentially allow such discrimination by hybridizing with any parasite DNA if it is present in the infected animal.

Radiolabeled probes have been used in helminth parasites to provide information on host–parasite relationships in mammalian host. In order to determine the progressive migration of cercariae of *F. hepatica* in the mammalian host, metacercariae were labeled with ^35^Se by exposing the infected snail host to ^75^Se-methionine. Mice were inoculated with labeled metacercariae and their distribution in the gut, body cavities, and liver was determined by autoradiography (Hsu 1986). In similar studies, cercariae of *S. bovis*, *Schistosoma intercalatum*, and *Schistosoma haematobium* were labeled with ^75^Se by exposing the intermediate snail host to the probe for 20 h in order to determine the relationships between the cercariae and schistosomulae (Christensen 1983). The cercariae retained the label for at least 32 days. For *Haemonchus contortus*, infective larvae were labeled by adding ^75^Se to fecal sediment containing the infective larvae. Labeled worms could be identified by autoradiography for up to 37 days after infection (Georgi and Le Jambre 1983). Glycoproteins of *S. japonicum*, *S. mansoni*, and *S. haematobium* that were genus or species specific were identified by labeling schistosomes with ^35^S-methionine (Nordena and Strand 1984). Characterization of genomic DNA for several species of nematode parasites of cattle provided potential diagnostic probes for differentiation of infections in cattle. DNA probes prepared from *Haemonchus placei*, *Ostertagia ostertagi*, *Cooperia oncophora*, and *Oesophagostomum radiatum* were able to detect as few as 25 eggs in fecal samples from infected cattle (Christensen et al. 1994).

Infective larvae and adult worms of *T. spiralis* were labeled with ^35^Se-methionine to determine the presence of stage-specific proteins in of both secreted and somatic antigens of different life-cycle stages of the parasite to identify proteins that might prove useful for diagnosis (Parkhouse and Clark 1983).

Microscopic examination of blood smears for piroplasms is the most widely used diagnostic method for detecting acute infections with *Theileria* spp., but is inadequate for detecting chronic infections in carrier animals where the parasitemia is low, resulting in false-negative diagnosis. A more reliable and sensitive detection method is to employ a specific theilerial antigen in PCR; a primer derived from a *T. parva*-specific 104-kDa antigen (p104 gene) labeled with ^32^P was able to detect as few as 1.4 parasites per microliter of blood (Odongo et al. 2010). For detection of *Theileria sergenti* infection, a genomic DNA library of *T. sergenti* was screened to detect clones containing the parasite's DNA sequences; two positive DNA inserts were purified from the recombinant plasmids and used as probes labeled with ^32^P. The probes were sensitive enough to detect 15 pg of purified *T. sergenti* DNA (equivalent to 1,200 parasites), while in diluted red blood cells infected with *T. sergenti* they were able to detect 8,000 parasites (Kajiwara et al. 1990).

This high level of discrimination has been used to distinguish between different *Theileria* species including *T. parva*, *T. annulata*, *Theileria mutans*, *Theileria buffleli*, and *Theileria taurotragi* (Allsopp et al. 1993). Six ^32^P oligonucleotide probes were designed that enabled unequivocal species-specific identification by detection of parasite subunit ribosomal RNA. The test also led to identification of previously unknown species of *Theileria* spp. in buffalo and sable antelope. The internal transcribed spacers (ITS) of several *T. parva* isolates were sequenced in order to distinguish between parasites that cause East Coast fever (*Theileria parva parva*) and those that cause corridor disease (*Theileria parva lawrenci*). Six oligonucleotides labeled with ^32^P were used in slot blots to detect parasites from cattle and buffalo. *T. parva parva* isolates hybridized with only a few of these probes, whereas *T. p. lawrenci* were more variable and hybridized with many more of them (Collins and Allsopp 1999). It was possible to characterize and isolate as *T. p. parva* with >90% probability or as *T. p. lawrenci* with >80% probability, and the probes could be useful in determining the distribution of different genotypes and subspecies of *T. parva*. DNA probes have also been used to characterize the vaccine that is used in East, Central, and Southern Africa to protect cattle against *T. parva*. The vaccine, known as the Muguga cocktail, comprises three stocks of *T. parva*, Muguga, Kiambu 5, and Serengeti-transformed. Four radiolabeled nucleic acid probes were used to determine the relationships among the three stocks of the vaccine (Bishop et al. 2001), the first attempt to comprehensively characterize the vaccine. There was close similarity between the Muguga and Serengeti stocks whereas the Kiambu 5 stock was significantly different from them. In view of these findings, it was suggested that only one of the Muguga and Serengeti-transformed stocks was essential for vaccination. However, later studies indicated that the two stocks were extremely diverse; they each comprised eight genotypes whereas Kiambu 5 showed no diversity. The Serengeti stock contained two of the genotypes present in Muguga as well as Kiambu genotype (Patel et al. 2011).

For *Babesia* infections, the requirements for diagnosis are related to the different stages of infection. During the early stage of infection, the parasitemia is low, and it is not easy to detect the parasite in the low number of infected erythrocytes. During the acute phase of infection, it is easy to identify infected animal by light microscopy, but it might also be necessary to differentiate between different hemoprotozoa to ensure appropriate chemotherapy. After treatment and recovery, it is necessary to monitor the effectiveness of treatment, and finally, there is a stage lasting several months when an animal becomes a “carrier” where antibodies are present but the parasitemia is extremely low. Detection of *B. bigemina* using conventional microscopy lacks sensitivity, as a large number of parasites (0.01% parasitemia) must be present to enable reliable detection. The problem of diagnosis is even more problematic in animals that have had a primary infection and then recovered, whereupon they harbor microscopically undetectable, sub-clinical infections. As these animals serve as reservoirs of infection, it is necessary to identify them as simply and speedily as possible. Originally, the only method available for their identification was to inoculate blood from the suspect animal and inoculate into splenectomized calf and examine it for signs of infection. Oligonucleotide probes selected from the SSrRNA of *B. bigemina* were therefore developed to use in the sensitive detection of the parasite in bovine blood (Reddy and Dame 1992). The probes were labeled with ^32^P and their sensitivity and specificity to detect parasite RNA assessed; they were able to detect *B. bigemina* RNA at levels of 0.012 fmol and showed no cross-reactivity with *B. bovis*, the parasite most likely to compromise clinical assessment of infection. When used to detect *B. bigemina* in bovine blood, the probes detected 100 parasite-infected red cells in 20 μl of bovine blood. In calves experimentally infected with 1,000 *B. bigemina* infected bovine erythrocytes, positive signals were obtained from blood samples collected from the infected animals from day 2 after inoculation. In contrast, parasites were not detected in the blood until 8 days or more after infection. A ^32^P-labeled DNA probe of *B. bigemina* was also found to hybridize with strains of the parasite from Texas, Mexico, Puerto Rico, Costa Rica, and Kenya (Buening et al. 1990). The probe hybridized with 10 pg of *B. bigemina* DNA and 150 infected erythrocytes and was useful for detecting carrier animals. For *B. bovis*, DNA probes were constructed and those that specifically hybridized to *B. bovis* DNA were selected for testing (Petchpoo et al. 1992). A probe with the highest sensitivity detected 25 pg of purified *B. bovis* DNA and as few as 300 parasites in 10 μl of infected blood. The probe did not cross-hybridize to *B. bigemina*, *Trypanosoma evansi*, *Plasmodium falciparum*, *A. marginale*, *Boophilus microplus*, and cow DNA. In the Southern blot analysis of genomic DNA, the probe could differentiate between isolates of *B. bovis* from different geographic locations. Vaccine strategies for *B. bovis* include the use of attenuated live parasites and various inactivated preparations. Although the attenuated vaccine provides the best protection against challenge with both homologous and heterologous strains, it has a number of disadvantages, including a short shelf-life, variation in virulence, contamination with host erythrocytes, and perpetuation of the life cycle by creation of a carrier state. Protective immunity in babesiosis may be directed against surface antigens associated with the merozoite stage. Possible candidate merozoite surface antigens of *B. bovis* have been identified by immunoprecipitation of ^125^I-labeled proteins with immune bovine serum and monoclonal antibodies (Goff et al. 1988a).

A quantitative solid-phase radioimmunoassay (RIA) was developed for the purpose of ranking sera from exposed animals according to their titer of anti-*B. bovis* antibody. The antigen was sonicated lysed infected blood cells, and antibody binding was detected with ^125^I-labeled anti-bovine IgG. A high specificity for *B. bovis* was demonstrated. There was good agreement in identifying exposed cattle when the test was compared with an indirect fluorescent antibody, and the RIA was more effective in detecting changes in antibody titer (Kahl et al. 1982).

Radiolabeled probes have also been used in studies on the immune responses to infections with pathogenic trypanosomes. The involvement of antibodies, macrophage activation, and complement in destroying *T. brucei* in immune animals was determined by using parasites labeled with ^75^Se-methionine (MacAskill et al. 1980). The labeled trypanosomes were inoculated intravenously into mice that had been immunized by infection and drug treatment, and 1 h later their distribution in various tissues and organs was determined. Destruction of the trypanosomes took place largely in the liver by antibody-mediated phagocytosis. In contrast, when experiments were conducted in mice with acute fulminating infections, no hepatic uptake was observed (MacAskill et al. 1981). It was concluded that the mice produced insufficient levels of antibodies to opsonize the extremely high numbers of trypanosomes present in the circulation since opsonization was observed when mice were inoculated with a trypanosome strain that caused a more chronic infection (MacAskill et al. 1981). A similar approach was used to determine the basis for trypanotolerance shown by certain breeds of cattle. Experimental studies were undertaken in two breeds of mice, one A/J that was fully susceptible to infection with *T. congolense* (MacAskill et al. 1983) and one, C57Bl, which was tolerant to infection. When the hepatic uptake of ^75^Se-labeled *T. congolense* by infected mice was measured as an index of antibody production, it was found that only C57B1 mice could remove circulating labeled parasites, this ability persisting for several weeks after infection. It was concluded that the trypanotolerant strain of mice depended on its more efficient antibody response to resist the effects of trypanosome infection (MacAskill et al. 1983). In other studies, a radioisotopic technique utilizing ^3^H-uridine was developed to determine the tissue distribution of intravenously inoculated *T. brucei* in susceptible (Swiss–Webster) and resistant (deer) strains of mice. During the initial 24 h of infection with labeled organisms, trypanosomes were cleared from the bloodstream of deer mice to a significantly greater extent than in Swiss–Webster mice Anderson and Banks 1982).

More informed analysis of the susceptibility of trypanotolerant livestock to infection was undertaken by comparing the humoral immune responses of N’dama (trypanotolerant) and Zebu (susceptible) cattle using immunoprecipitation of proteins from ^35^S-methionine and ^35^S-cystein labeled trypanosomes (Shapiro and Murray 1982). Three proteins of MW 110,000–300,000 were recognized by cattle that were able to control infections and the trypanotolerant N’dama were able to recognize more of the trypanosome proteins than the Zebu cattle. Immune responses in cattle infected with two different clones of *T. brucei* and antibody response was followed by radioimmunoassay. The variable surface glycoproteins (VSGs) of the two clones was labeled with ^125^I and precipitated with immune sera (Musoke et al. 1981). There were at least two peaks of antibody activity to the infecting clones, with the second peak much higher than the first. IgG1 and IgM antibodies were eluted from immunoabsorbent columns on which the VSGs were coupled for use in neutralization tests. IgM antibodies from the first peak of antibody activity were more efficient in neutralizing trypanosomes than IgGl, but the reverse was true of the antibodies isolated from the second peak. Similar radioimmunoassays in *T. brucei*-infected wildebeest (Rurangirwa et al. 1986) showed a typical sequence of IgM, IgG1, and IgG2 antibodies, with a higher frequency of occurrence than seen in cattle. Trypanosomes were also metabolically labeled with ^35^Se-methionine to determine the interaction between immune serum and peripheral blood leukocytes. Sera from wildebeest had a higher propensity to induce adherence to both homologous and heterologous leukocytes.

### Radiolabeled probes and radioimmunoassays for the diagnosis and characterization of microbial infections

*A. marginale* is a rickettsial hemoparasite of cattle that is transmitted by biological vectors (ticks) and mechanical vectors such as biting flies. The acute phase of disease causes severe anemia, weight loss, abortion, and death, but animals that recover are persistently infected with low levels of organisms that cannot be detected microscopically. These carrier animals act as disease reservoirs and are responsible for causing continual disease outbreaks. In order to understand the role that carrier animals play in disease transmission, it is necessary to use tests that are more effective than serological diagnosis in order to discriminate between active carriers and animals that merely harbor antibodies due to a prior infection; a number of nucleic acid probes have been identified that provide the means to achieve this. A ^32^P-labeled nucleic acid probe derived from a gene encoding a surface protein of *A. marginale* proved specific for the parasite, showing no evidence of cross-hybridization with other hemoparasites (Eriks et al. 1989). This probe detected 0.01 ng of genomic DNA and 500–1,000 infected erythrocytes in 0.5 ml of blood. Tests with animals that were known to be carriers showed that parasitemia levels varied considerably in individuals, suggesting that animals probably differed significantly in their ability to transmit infection. The same probe was also useful in identifying infections in adult and larval stages of the ticks *Dermacentor variabilis* and *Dermacentor andersoni* that had been infected as nymphs or adults (Goff et al. 1988b). An inhibition radioimmunoassay based on a common-stage 36-kDa antigen was used to detect *Anaplasma* in individual or pooled infected ticks and as a sensitive quantitative assay for characterization of vertebrate and invertebrate infection (Palmer et al. 1985). An improved method for isolation of genomic DNA was used to label with ^32^P a recombinant probe that showed 100% specificity and sensitivity by hybridization in dot blotting and Southern blotting; it was able to detect 500–1,000 infected erythrocytes per microliter, corresponding to a parasitemia of 0.01% (Aboytes-Torres and Buening 1990). Cloned DNA probes labeled with ^32^P have also been used to identify goats infected with *A. ovis* (Shompole et al. 1989). The DNA probe detected a parasitemia of 0.0034% in infected blood, a sensitivity that was suitable for use in surveillance and epidemiological studies.

*Cowdria (Ehrlchia) ruminantium* is another rickettsia that causes serious losses to ruminants in sub-Saharan Africa, where it is transmitted by ticks of the genus *Amblyomma*. Demonstration of the parasite in live animals or detecting them in ticks is difficult, requiring inoculation into susceptible ruminants. The parasite is not detectable by light microscopy of stained blood smears, and it requires examination of endothelial cells from biopsy or post-mortem specimens of brain tissue. A ^32^P-labeled oligonucleotide of a clone known as pCS20, derived from a Zimbabwean strain of *C. ruminantium*, detected *C. ruminantium* DNA in adult ticks that had been allowed to feed as larvae on infected goats, in nymphs that had fed as larvae on infected goats and in adult ticks that had fed as nymphs on infected goats (Waghela et al. 1991). The probe was also used to detect *C. ruminantium* in the midguts of male and female *Amblyomma hebraeum* ticks, the principal vector of the parasite in southern Africa (Yunker et al. 1993). The probe also hybridized with DNA of four heartwater strains from Zimbabwe, two from South Africa, one from Nigeria, and another from Guadeloupe in the Caribbean (Mahan et al. 1992). *C. ruminantium* DNA was also detected in infected sheep before any febrile reaction had been seen, the first clinical sign of disease. The hybridization signal varied, probably depending on the level of parasitemia in the host (Mahan et al. 1992). A radioimmunoassay has been described for the detection of *A. marginale* antibodies in cattle sera (Schuntner and Leatch 1988). Optimal sensitivity and specificity were obtained by using two antigens, an *A. marginale* antigen and an RBC antigen (obtained before infection was established) from the same calf. In addition, sera were pre-absorbed with RBC from healthy cattle and with sonicated *B. bovis*. Of 86 sera obtained from cattle with proven *A. marginale* infection, 85 had positive results by use of this test. Of 100 sera obtained from cattle raised in an anaplasmosis-free area, 98 yielded negative results, and sera obtained from 35 cattle (97 sera) infected with *B. bigemina* and from 18 cattle infected with *T. orientalis* yielded negative results. In *B. bovis-*infected cattle, 99 of 100 sera were negative for *A. marginale*. *A. marginale* antibodies were detected in 18 cattle for 2 years after natural infection. The sensitivity and specificity of the test were each determined to be 98.8%.

Definitive diagnosis of brucellosis can be made by isolation and identification of the causative organism. However, a considerable number of serological test have been developed, including primary binding assays such as RIA that have however not found wide application for routine use. RIA utilizing ^125^I-labeled immunoglobulins was able to detect both IgG_1_ and IgG_2_ subclass antibodies to *B. abortus* in bovine serum, but did not react with IgM class, suggesting that it might be possible to distinguish between infected and vaccinated cattle (Chappel et al. 1972). In measuring the serological response in cattle vaccinated against *B. abortus*, the RIA gave positive reactions in many cases where the CFT was negative, suggesting that the RIA was more effective detecting brucellosis (Chappel et al. 1982). In experimentally infected cattle, RIA was most sensitive in cattle that had been vaccinated and gave fewer false-negative reactions than other serological tests (Hayes and Chappel 1982). A solid-phase RIA, utilizing 96-well microtiter plates coated with antigen and ^125^I-labeled staphylococcal protein A for detection, was considered sensitive and able to discriminate between false positives and false negatives (Lawman et al. 1984). Antibody detection was 16 to 32 times more sensitive than CFT and 4- to 64-fold more sensitive than the standard tube test titer. An RIA to detect class-specific antibodies to *B. abortus* was developed using antigen bound to glass tubes and then detecting positive reactions by binding bovine antibodies with rabbit anti-immunoglobulin antiserum followed by incubation with ^125^I-labeled sheep anti-rabbit immunoglobulin (Levieux 1978). This three-stage technique gave best discrimination between sample and background counts and was some 10,000 times more sensitive than CFT.

*B. anthracis* can be readily isolated from blood or tissues of a recently dead animal that died of anthrax, and the morphology of *B. anthracis* on blood agar is quite characteristic; visualization of the bacillus in a blood smear stained with methylene blue is fully diagnostic.

Serological tests of infected animals are rarely used for diagnostic purposes, although there is an ELISA and a PCR is also available. Nevertheless, immunoradiometric (IRMA) assays have been used as potential diagnostic tools. A solid-phase IRMA in which *B. anthracis* spores labeled with ^3^H were used in direct and indirect tests were heat fixed on slides (Phillips and Martin 1983). In a modification of the test, ^125^I-labelled protein A was used in an indirect IRMA (Phillips and Martin 1984). Neither of these approaches was significantly outstanding due to erratic results and high background noise.

Leptospirosis is a widespread zoonotic disease caused by pathogenic strains of the genus *Leptospira* which are capable of infecting most mammalian species. Infection occurs either through direct contact with an infected animal or indirect contact with contaminated soil or water. In livestock, the disease causes economic losses due to abortion, stillbirth, infertility, decreased milk production, and death. The detection of leptospira in biological fluids is important for the diagnosis of leptospirosis in animals; however, the sensitivity of dark field microscopy, the usual detection method, is often inadequate. Several serological techniques have been developed to increase the sensitivity of diagnosis including some using radiolabeling. An inhibition RIA was developed using serovar-specific main TM antigen of *L. interrogans* labeled with ^3^H sodium borohydride. There was no cross-reactivity with other serovars of *Leptospira* and the assay was more sensitive than CFT (Chappel et al. 1985). An enzymatic radioimmunoassay has been developed for detecting *Leptospira interrogans* serovar *pomona* in porcine urine (Chappel et al. 1985). A solid-phase RIA to detect leptospires or their antigens in urine samples did not show a high sensitivity (Bahaman et al. 1986). DNA extracted from *L. interrogans* serovar *pomona* was labeled with ^32^P and used as a genomic probe to detect leptospiral DNA. The sensitivity of detection was 160 pg of leptospiral DNA or 1.1 × 10^3^ leptospires, and assays with nylon membranes were somewhat more sensitive than assays with nitrocellulose membranes (Millar et al. 1987).

Foot and mouth disease is of major concern to the livestock industry in both developed and developing countries. Strict import measures have been adopted by many countries to prevent introduction of the disease, and various diagnostic tests have been developed to enable rapid and sensitive detection of cases of the disease and for typing the different virus serotypes. A number of radioimmunoassays were developed to aid diagnosis and differentiation, but none of them has been adopted in practice. A radioimmunoassay technique was used to compare different samples of type SAT 2 FMD viruses by measurement of the competition of heterologous virus with homologous virus for previously titrated homologous antiserum. Viruses could be grouped according to their ability to compete with the homologous virus, and statistically significant differences between virus “groups” were observed. The results of a comparison of the relationships between the viruses using RIA and complement fixation tests did not always correlate (Crowther et al. 1979). RNA competition hybridization using ^32^P-labeled RNA and competitive radioimmunoassays with ^35^S-labeled viruses were used to compare the biochemical and serological characteristics of five isolate of FMD, serotype A (Robson et al. 1979). Three A_22_ viruses were found to be different from A_5_ and A_24_ viruses by RNA hybridization. Similar findings were obtained with RIA. A rapid, reproducible solid-phase radioimmunoassay was developed for the detection of antibodies against a specific region of the structural protein VP1 of the A_24_ and 01 serotypes of FMD virus. The RIA antibody titers showed a positive correlation with neutralizing antibody titers determined by a mouse protection assay (Patzer et al. 1985). Competition RIA using ^35^S-labeled viruses was used to define four antigenic determinants in the virus capsid. One determinant detected on the intact particle was considered to be of epidemiological importance since it was present on the intact virion, was found to vary in heterologous field strain, induced neutralizing antibody responses, and was important in the immunogenicity of the virus (Haresnape and McCahon 1983). Recombinant complementary DNA (cDNA) probes labeled with ^32^P were used to detect and differentiate between FMD viruses (McFarlane et al. 1990). It was possible to differentiate between FMD virus serotypes A, O, and C, using cDNA probes from individual serotypes corresponding to protein VP1. The assays appeared to be more sensitive than complement fixation tests (CFT) and ELISAs, being able to detect 1 pg of virus.

African swine fever (ASF) viruses produce a range of symptoms in domesticated and wild pigs varying from acute to chronic disease and apparently healthy virus carriers. The presence of ASFV antibodies is indicative of infection and as they persist for long periods, they are a good marker for diagnosis of the disease. Enzyme immunoassays, immunoblotting, and indirect fluorescent antibody tests have all been used in diagnosis. Solid phase RIA using microtiter plates and ^125^I-labeled IgG has been used to detect both viral antigen and antibody (Crowther et al. 1979) and was shown to be 1,000 times more sensitive than an immune-electro-osmophoresis test for detection of ASFV antibody. RIA detected antibodies within 3–4 days of infection, in contrast with the gel diffusion tests that was not positive until 10 days post-infection (Wardley and Wilkinson 1980). An indirect RIA was also considered to be a possible candidate for diagnosis of Aujeszky’s disease in pigs in the field, as it was found to be highly sensitive in detecting infection, and was also more rapid than a virus neutralization tests Rodak et al. 1981). It could be completed in 1 or 2 days, it did not require the preparation of cell cultures and the use of aseptic conditions, and properly stored control material could be stored for up to a year without decline in effectiveness. With its high specificity and sensitivity, it was considered more reliable in detecting infected pigs earlier and more accurately than virus neutralization tests.

For Rift Valley fever, a radioimmunoassay using ^125^I-labeled protein A was not as sensitive as other tests including neutralization tests or hemagglutination inhibition assays to detect antibodies in infected sheep, probably due to the poor binding of protein A to sheep immunoglobulins (Swanepoel et al. 1986).

An indirect micro-radioimmunoassay to detect antibodies in chickens was developed for Newcastle disease using radioactively labeled rabbit anti-chicken Fab and virus-infected microcultures of chick embryo fibroblasts. The Newcastle disease virus-infected micro-cultures were formalin fixed and stored at 4°C for up to 4 months without affecting the sensitivity of the test. The micro-technique was found to be a highly sensitive and specific assay for anti-viral antibody (Cleland et al. 1975). Other RIAs utilized a radiolabeled membrane fraction protein, 150,000 MW to detect both antibodies and antigens of Newcastle diseases virus (Spira et al. 1976). Antibody titers were as high as 1:51,200 by indirect RIA and in an inhibition RIA 5 ng of viral protein could be detected. The labeled antigen could be stored for at least 6 weeks at −20°C without loss of activity.

In southern Africa, widespread infections with a virus known as equine encephalosis virus (EEV) appeared in 1967 that became a serious disease, often causing epidemics (Viljoen and Huismans 1989). The morphology, clinical symptoms, and pathology of this disease in horses resemble infections caused by African horse sickness virus (AHSV) thereby making diagnosis difficult. In order to obviate this problem, a number of genome-specific probes were developed that would assist in differentiating the two viruses. Different ^32^P-labeled EEV serotype-specific probes were prepared and two of them of genome segments 3 and 5 were able to differentiate between EEV and AHSV (Viljoen and Huismans 1989). A reverse transcription polymerase chain reaction (RT–PCR) based on the gene encoding the NS2 protein of AHSV was developed for rapid detection of AHSV. The specificity of RT–PCR products was confirmed by Southern blot hybridization using a radioactively labeled cDNA probe specific for the NS2 gene. This RT–PCR could discriminate between all known members of the AHSV and equine encephalosis virus serogroups. In an immune horse which had been vaccinated with an AHSV (serotype 4) VP2 subunit vaccine, viral RNA could be detected for up to 22 weeks post-challenge. AHSV RNA was detected in various organs of an infected horse. Viral RNA was also detected by RT–PCR in nine suspected field cases of African horse sickness in which virus was isolated from eight (Bremer and Viljoen 1998). Several ^32^P-labeled genomic probes of bluetongue virus, epizootic hemorrhagic disease virus, and EEV were used to detect virus-specified RNA in infected cells. Probes derived from the genome segment that encoded the non-structural protein NS1 were the most sensitive, detecting RNA within 2–3 h of infection (Venter et al. 1991).

### Stable isotopes

In a world where the movement of animals or their products is commonplace and in which changes in environment due to climate change can potentially affect the spread of infectious diseases and their vectors, there is a critical need for technologies that will enable identification of the geographical origin of those diseases and, where necessary, provide information on the feeding habits and associated movements of their vectors. A technique that has been extensively used in studying the ecology of animals, stable isotope analysis (SIA), is likely to be in the forefront of methods that will be most useful in providing the means to understand the epidemiology of diseases. Stable isotopes are the naturally occurring forms of elements with differing nuclear masses and, as the name implies, they do not undergo radioactive decay; many elements have one or more stable isotopes and there are over 250 in existence. However, for the purposes envisaged, it is only necessary to concentrate analysis on a small number of isotopes that are involved in important biological and ecological processes. The isotopes are measured by mass spectrometry as isotopic differences relative to international standards prepared by IAEA and reported as ratios in delta (δ) units as parts per thousand. The value of SIA is based on the strong correlation between levels of certain isotopes in the environment and the concentration of the same isotopes in animal tissues. A number of isotopes can be usefully employed and fall into two categories: those of low atomic mass, carbon (C), nitrogen (N), sulfur (S), oxygen (O), and hydrogen (H); and those of high atomic mass, strontium (Sr) and lead (Pb). The heavy isotopes are representative of environmental processes and can be used to trace uptake from soils.

The presence of the light stable isotopes in animal tissues depends on both biological and environmental processes. For example, stable carbon (δ^13^C) and nitrogen (δ^15^N) participate in biochemical processes in fixation in plants, while δ^13^C found in tissues reflects dietary intake, and δ^15^N in tissues is often affected by water and nutritional stress. Stable sulfur (δ^34^S) varies widely in aquatic and terrestrial environments and reflects sources of nutrients in food webs. For animal movements, the isotopes of most interest are hydrogen (δ^2^H) and oxygen (δ^18^O) ratios in tissues that accurately reflect those in lakes, rivers, oceans, and in groundwater. Using stable isotopes to characterize a population involves examining the isotopic signatures of a few individuals that are representative of the entire population. Studies have been in progress for several years using stable isotopes to characterize and differentiate among animal populations (particularly birds) using δ^13^C and δ^15^N values in metabolically active tissues (blood and muscle), but presently, the most effective tracers appear to be the hydrogen isotopes found in metabolically inert, seasonally grown tissues, such as feathers and claws. Feathers retain this information until replaced or molted, typically once per year. Conversely, claws are continuously growing and can theoretically provide a time-integrated profile depending on claw growth rates. Once the isotope profile of a particular bird population is known, any individuals from the population can provide information on global migration of that species.

The hydrogen and oxygen isotope composition of environmental water varies spatially across the globe, and because it is a constituent of many biosynthetic pathways, the isotopes’ presence is relayed to animal tissues, providing the means to link precipitation isotope values with those in biological tissues. Global grids of hydrogen and oxygen water isotopes have been constructed to provide accurate estimates of δ^2^H and δ^18^O in water that can then be compared to animal samples of known or unknown origin. These grids can be constructed using the data from the Global Network for Isotopes in Precipitation database collated by the IAEA.

### Migratory connectivity and emerging zoonoses

Migratory connectivity is the term used to describe the relationship between populations of animals (especially birds) and geographic locations at different time points during the year. Migratory connectivity studies are particularly useful for tracking bird-borne diseases; however, traditional methods of banding and recapture provide limited details about the migratory patterns of a species of bird because few banded individuals are ever captured twice and GPS tracking devices are expensive and can be used on only small numbers of individual animals.

It is generally acknowledged that the majority of emerging infectious diseases are zoonotic in nature and that most of them are derived from wild animals. There is a need to understand the different factors which increase the risk of disease incursion and spread in relation to contact between animal, human, and wildlife populations brought about by trade and animal migration. The global threat from emerging infectious diseases to domesticated livestock and humans posed by long-distance movement of migratory wild animals is particularly serious.

The ubiquity of ticks and their importance in the transmission of pathogens involved in human and livestock diseases is well known. However, studies on tick–bird systems have mainly focused on land birds; for example, birds can harbor *Ixodes* ticks infected with *Borrelia* spp. that are a cause of Lyme disease in the USA (Reed et al. 2003). However, the role of seabirds in the ecology and epidemiology of tick-borne pathogens is rarely considered. Seabirds typically have large population sizes, wide geographic distributions, and high mobility, which make them significant potential players in the maintenance and dispersal of disease agents at large spatial scales. They are parasitized by at least 29 tick species found across all geographical regions of the world and can harbor a large diversity of pathogens, although there have been no detailed studies of this diversity (Dietrich et al. 2011). Birds are also reservoirs of infection and also implicated in the dispersal of West Nile Virus by the bites of mosquitoes. Other common pathogens in wild birds are *Salmonella typhimurium* and *Campylobacter* spp. which they acquire during their feeding and can be a source of infection for domesticated poultry and humans (Reed et al. 2003).

Of most concern however is the threat of Highly Pathogenic Avian Influenza (HPAI) that has caused devastating economic losses globally for the poultry industry, as well as being a potentially serious zoonotic disease of humans. Migratory waterfowl appear to play a role in the dissemination of HPAI, as there appears to be an association with infections in wild birds and outbreaks of disease in domesticated poultry (Gaidet et al. 2010; Takekawa et al. 2010), but the picture is not clear. It would be useful therefore to reveal migration patterns and enable identification of the breeding areas of birds sampled in intermediate stopover sites, or non-breeding grounds, and in samples collected from disease outbreak sites. Since the beginning of the HPAI epizootic, over 100 bird species from 13 orders have been found to be infected with the H5N1 AI virus. Despite this knowledge, little is actually known about the prevalence of HPAI in birds or how it is transmitted through avian populations via migratory routes. Indeed, there have been few studies on how mass movements of wild animals in seasonal migration affect the transmission and evolution of pathogens within a population and the infection risk to other species. Investigations utilizing satellite tracking devices have not unequivocally linked HPAI in wild birds with the infections occurring in poultry. Hence, there have been recommendations that SIA should be added to banding and satellite tracking technologies as part of the surveillance strategy for HPAI (Chang et al. 2009).

Although SIA has been used extensively in the last 20 years for the study of long-distance dispersal and ecology of migratory birds, especially in North America and Europe (e.g., Hobson 2005, 2008, 2011; Hobson et al. 2009, 2010; Van Wilgenburg and Hobson 2011), its application in studies on animal migration and infectious disease risk has not been adequately addressed. In addition, since many of the incidents involving HPAI have occurred in wild birds using flyways that originate in Asia, the use of SIA to understand the epidemiology of the disease will be particularly challenging. The use of SIA has already shown promise for establishing the origins of birds from Mongolia (Pérez et al. 2010). The values of δ^2^H in actively growing feathers agreed with those expected from the isotopic values found in the collection sites. There is still need to improve the data on δ^2^H isoscapes in the region as these are not well described at present (Chang et al. 2009) as well as development of a feather collection protocol to standardize sampling efforts and develop an Asian δ^2^H feather isoscape base map (Pérez et al. 2010). Stable hydrogen isotope analysis has also been used to determine the biogeography of avian hematozoan infections caused by *Leucocytozoon* and *Haemoproteus* spp. in birds of prey (Smith et al. 2004) to establish the place of origin of the infected birds.

### Establishing host–parasite feeding preferences

Identifying the roles of reservoir hosts and the vectors of animal disease is a major challenge in epidemiological studies and stable isotopes can provide insights into the ecology of vector-borne diseases. Molecular methods of blood meal typing are sensitive, but can be compromised by rapid digestion of the blood, denaturing of DNA, and mixed blood meals (Gómez-Díaz and Figuerola 2010). Stable isotopes represent a complementary technique that can avoid these problems. The isotopic composition of vector tissues is a function of the isotopic signature of each host species on which it fed and also of the relative proportions of each host species assimilated. Hence, once the isotope ratios measured in an arthropod vector are known, inferences regarding its diet and feeding behavior can be made (Hood-Nowotny and Knols 2007). Determination of carbon (δ^13^C) and nitrogen (δ^15^N) isotope ratios is the most widely used technique for characterizing feeding relationships among vectors/hosts, but the inclusion of other stable isotopes, such as hydrogen, oxygen and sulfur, can make it easier to differentiate between potential hosts in diverse communities. In order to be effective, the determination of discrimination factors requires the development of a library of isotopic signatures of the possible hosts of the vector in the area being studied. The value of SIA in resolving the roles of different hosts and vectors in the ecology of multi-host pathogens has been shown by the studies of Stapp and Salkeld (2009) on the host–parasite relationship between mice, prairie dogs, and fleas in the transmission of *Yersinia pestis*. It has been assumed that the mice spread the bacterium among prairie dog colonies by sharing the flea of the prairie dog, *Oropsylla hirsuta*. Stable nitrogen analysis showed that the fleas did not feed on the mice and that they became infected by other methods, such as mechanical transmission or scavenging carcasses. Feeding on vertebrate blood is a critical stage in the transmission of numerous pathogens by ticks, mosquitoes, tsetse flies, midges, and other hematophagous arthropods, but identifying the sources of their blood meals by conventional techniques is only useful for about 36 h after they have fed. In contrast, carbon and nitrogen stable isotope profiles of mosquitoes fed up to 7 days previously could be used to identify successfully the mammal hosts on which the insects had fed (Stapp and Salkeld 2009). Proof-of-concept studies have also demonstrated that stable isotope ratios of nitrogen and carbon of host blood are was detected in unfed nymphal *Ixodes ricinus* that had developed from larvae fed on that host. SIA of unfed ticks was considered to have potential for determining the physiological age of unfed ticks, identifying the season in which the previous stage had fed, and for identifying the main hosts utilized by ticks (Schmidt et al. 2011). Stable isotopes have also been used in determining relationships between the animal host and the vector. For example, the vampire bat, *Desmodus rotundus*, is a significant threat to animals and humans as it is able to transmit pathogens such as the rabies virus, but the host selection preferences are not well understood. The stable carbon isotope signatures of the blood ingested by the bats were used to identify the hosts on which the bats were feeding. Cattle ingest grass and exhibit the C_4_ metabolic pathway of CO_2_ fixation, whereas other mammals in the forested areas feed on plants that show the C_3_ pathway. In an area where both cattle and forest animals were living, the bats preferentially fed on cattle, possibly because they represented an easy source of food since they were herded together and easy prey for the bats (Voigt and Kelm 2007). Clearly there is a significant role for the use of stable isotopes in this sort of study, but their use in understanding host–parasite interactions in vector-borne diseases is still largely unexplored.

### Characterization of microorganisms

Studies of stable isotopes have unequivocally demonstrated that the isotope ratios in animal tissues are a function of the food and water that they consume and can therefore be related to the geographic origins of those animals thereby providing a means of determining origin in migratory animals. In the same way, there is substantial evidence to indicate that stable isotope ratios of microorganisms that are maintained *in vitro* are also a function of the nutrients and water that are used in the preparation of culture media (Kreuzer-Martin et al. 2004a, b). A survey of carbon, nitrogen, and hydrogen stable isotope ratios in over 500 samples of bacteriological culture media used to produce *Bacillus subtilis* showed that they varied according to the isotopic variation in the plant and animal products upon which the media were based (Kreuzer-Martin et al. 2004b). The variation was sufficient to translate into substantial isotope variation in cultures grown on different batches of media. This finding has potentially important application in the field of microbiology where different bacteria or viruses are cultured and possibly used in vaccine production. By determining the stable isotope signatures of batches of organisms at the time of production, it would be possible to identify those products at a later date. Even if several manufacturers provided genetically identical organisms that would express the same genotype, they would not show the same isotope ratios. In this way, it would be possible to determine the origin of attenuated versions of organisms in vaccines, leading to more effective quality control of vaccines and allow closer monitoring of their distribution and use in the field.

### Disease diagnosis

An interesting perspective on the use of stable isotopes lies in their application in the diagnosis of diseases that lead to gastrointestinal malfunction and can be detected on the basis of non-invasive breath tests using stable carbon ^13^C. These have been used in human medicine and, to a limited extent, in studies on animals. In veterinary diagnostics, breath samples can be easily collected from animals by means of a face mask or collection chamber with minimal disturbance to the animal. Some 3,000 gases have been identified in exhaled human breath, and some of them have been linked with specific disease processes. Diagnosis is based on ingestion of a suitable substrate labeled with stable carbon relevant to the intestinal process being investigated, then analyzing breath samples by isotope ratio mass spectrometry. Applications for breath testing in humans include gastroenterology, hepatology, and oncology. *Helicobacter pylori* is a bacterium that induces inflammation of the gut causing chronic gastritis, ulcers, and can also lead to development of cancer. The bacterium survives in gastric acid by excreting large amounts of urease that breaks down any urea in the stomach to ammonia and carbon dioxide. Diagnosis can be made by ingesting urea labeled with ^13^C. The presence of *H. pylori* will be demonstrated by the production of large amounts of CO_2_ labeled with ^13^C (Graham et al. 1987). Hepatic function affected by hepatitis C infection can be quantified by a ^13^C-aminopyrine breath test (Armuzzi 1999). Preliminary experiments for collecting breath samples to measure intestinal dysfunction in chickens (Hughes et al. 2008) have shown promise and could be further developed to enable diagnostic applications. Hepatic disorders are a common problem in dogs and cats, and they are difficult to diagnose and may often require hepatic biopsy in order for confirmatory diagnosis. A ^13^C-aminopyrine test was developed for use in dogs in which instead of measuring labeled CO_2_ in the breath, it was extracted from the blood (Moeller et al. 2001). This was later validated and optimized for clinical application (Chiaramonte et al. 2003; DeBiasio et al. 2008). Horses often develop upper gastrointestinal tract dysfunctions due to harsh training and stress, and in the search for safe, non-invasive tests that are simple to perform, stable isotope technologies based on those applied in human subjects have been developed to determine solid-phase emptying of the gut (Sutton et al. 2003). The animals are fed a ^13^C-octanoic acid labeled meal; the label is not metabolized but is absorbed by the small intestine, then undergoes hepatic oxidation leading to production of ^13^C-labeled CO_2_ in the breath. The rate of appearance of labeled CO_2_ is a measure of the gastric emptying. Results confirmed that the test gave an accurate measurement of delayed emptying and could be used for investigations of a number of conditions such as gastric ulceration, grass sickness, and duodenitis (Sutton et al. 2003). Another test employed ^13^C acetic acid as the source of stable carbon and similarly showed potential for measuring intestinal problems in equines (Sasaki et al. 2005).

## Conclusions

It is clear that nuclear technologies have contributed significantly to livestock health research, and their refinement and development has led to methods for diagnosis that are of direct assistance to the farmer. Modern molecular techniques for diagnosis and characterization of DNA owe their development to the earlier use of radiolabeling to carry out the tests. The aim of this review is to enable an appraisal of these technologies and perhaps allow researchers to re-examine them and determine if they can complement what are regarded today as “state of the art technologies”. For instance, stable isotope analysis and molecular characterization can both be used in typing of strains of microorganisms, providing information on environmental parameters on the one hand and the genetic makeup of the organisms on the other. Stable isotopes can also be used to look at animal movements and could have application in addition to tracking the movements of wild birds by tracing outbreaks of transboundary diseases that have arisen from the movement of livestock or incursions of infected wildlife into livestock-rearing areas. It is essential that the research outcomes are translated to the farmer, thereby contributing to food security. A major challenge in the development of such new technologies is in ensuring that they are suitably modified so that they can be easily transferred to developing countries; the Joint FAO/IAEA Division will be in the forefront of enabling this to occur.
